# The Back Pain Consortium (BACPAC) Research Program Data Harmonization: Rationale for Data Elements and Standards

**DOI:** 10.1093/pm/pnad008

**Published:** 2023-01-31

**Authors:** Anna Batorsky, Anton E Bowden, Jessa Darwin, Aaron J Fields, Carol M Greco, Richard E Harris, Trisha F Hue, Joseph Kakyomya, Wolf Mehling, Conor O’Neill, Charity G Patterson, Sara R Piva, Nico Sollmann, Vincent Toups, Ajay D Wasan, Ronald Wasserman, David A Williams, Nam V Vo, Matthew A Psioda, Micah McCumber

**Affiliations:** Department of Biostatistics, Gillings School of Global Public Health, University of North Carolina at Chapel Hill, Chapel Hill, NC, United States; Department of Mechanical Engineering, Brigham Young University, Provo, UT, United States; Department of Physical Medicine and Rehabilitation, School of Medicine, University of Pittsburgh, Pittsburgh, PA, United States; Department of Orthopaedic Surgery, University of California San Francisco, San Francisco, CA, United States; Department of Psychiatry, University of Pittsburgh School of Medicine, Pittsburgh, PA, United States; Department of Physical Therapy, School of Health and Rehabilitation Sciences, University of Pittsburgh, Pittsburgh, PA, United States; Chronic Pain and Fatigue Research Center, Department of Anesthesiology, University of Michigan Medical School, Ann Arbor, MI, United States; Department of Epidemiology & Biostatistics, University of California San Francisco, San Francisco, CA, United States; School of Health and Rehabilitation Sciences Data Center, University of Pittsburgh, Pittsburgh, PA, United States; Department of Family and Community Medicine, University of California San Francisco, San Francisco, CA, United States; Department of Orthopaedic Surgery, University of California San Francisco, San Francisco, CA, United States; Department of Physical Therapy, School of Health and Rehabilitation Sciences, University of Pittsburgh, Pittsburgh, PA, United States; School of Health and Rehabilitation Sciences Data Center, University of Pittsburgh, Pittsburgh, PA, United States; Department of Physical Therapy, School of Health and Rehabilitation Sciences, University of Pittsburgh, Pittsburgh, PA, United States; Department of Radiology and Biomedical Imaging, University of California San Francisco, San Francisco, CA, United States; Department of Diagnostic and Interventional Radiology, University Hospital Ulm, Ulm, Germany; Department of Diagnostic and Interventional Neuroradiology, School of Medicine, Klinikum rechts der Isar, Technical University of Munich, Munich, Germany; TUM-Neuroimaging Center, Klinikum rechts der Isar, Technical University of Munich, Munich, Germany; Department of Biostatistics, Gillings School of Global Public Health, University of North Carolina at Chapel Hill, Chapel Hill, NC, United States; Department of Anesthesiology and Perioperative Medicine, School of Medicine, University of Pittsburgh, Pittsburgh, PA, United States; Back and Pain Center, University of Michigan, Ann Arbor, MI, United States; Department of Anesthesiology, University of Michigan, Ann Arbor, MI, United States; Chronic Pain and Fatigue Research Center, Department of Anesthesiology, University of Michigan Medical School, Ann Arbor, MI, United States; Department of Anesthesiology, University of Michigan, Ann Arbor, MI, United States; Department of Psychiatry, University of Michigan Medical School, Ann Arbor, MI, United States; Department of Internal Medicine-Rheumatology, University of Michigan Medical School, Ann Arbor, MI, United States; Department of Orthopaedic Surgery, University of Pittsburgh School of Medicine, Pittsburgh, PA, United States; Ferguson Laboratory for Orthopaedic and Spine Research, University of Pittsburgh, Pittsburgh, PA, United States; Department of Biostatistics, Gillings School of Global Public Health, University of North Carolina at Chapel Hill, Chapel Hill, NC, United States; Department of Biostatistics, Gillings School of Global Public Health, University of North Carolina at Chapel Hill, Chapel Hill, NC, United States

**Keywords:** data integration, harmonization, common data elements, low back pain, data standards

## Abstract

**Objective:**

One aim of the Back Pain Consortium (BACPAC) Research Program is to develop an integrated model of chronic low back pain that is informed by combined data from translational research and clinical trials. We describe efforts to maximize data harmonization and accessibility to facilitate Consortium-wide analyses.

**Methods:**

Consortium-wide working groups established harmonized data elements to be collected in all studies and developed standards for tabular and nontabular data (eg, imaging and omics). The BACPAC Data Portal was developed to facilitate research collaboration across the Consortium.

**Results:**

Clinical experts developed the BACPAC Minimum Dataset with required domains and outcome measures to be collected by use of questionnaires across projects. Other nonrequired domain-specific measures are collected by multiple studies. To optimize cross-study analyses, a modified data standard was developed on the basis of the Clinical Data Interchange Standards Consortium Study Data Tabulation Model to harmonize data structures and facilitate integration of baseline characteristics, participant-reported outcomes, chronic low back pain treatments, clinical exam, functional performance, psychosocial characteristics, quantitative sensory testing, imaging, and biomechanical data. Standards to accommodate the unique features of chronic low back pain data were adopted. Research units submit standardized study data to the BACPAC Data Portal, developed as a secure cloud-based central data repository and computing infrastructure for researchers to access and conduct analyses on data collected by or acquired for BACPAC.

**Conclusions:**

BACPAC harmonization efforts and data standards serve as an innovative model for data integration that could be used as a framework for other consortia with multiple, decentralized research programs.

## Introduction

The Back Pain Consortium (BACPAC) Research Program is a translational, patient-centered effort to address the need for effective and personalized therapies for chronic low back pain (cLBP).^1^ BACPAC consists of a Data Integration, Algorithm Development, and Operations Management Center (DAC); 3 Interdisciplinary Mechanistic Research Centers (MRCs); 4 Clinical Trial Centers (CTCs); 7 Technology Research Sites (Tech Sites); and a Consortium-wide Sequential Multiple Assignment Randomized Trial (SMART), henceforth referred to as the Biomarkers for Evaluating Spine Treatments Trial (BEST), focused on advancing knowledge of the etiology and treatment of cLBP as part of the National Institutes of Health (NIH) Helping to End Addiction Long-term^SM^ Initiative, or NIH HEAL Initiative. One of the aims of BACPAC is to develop an integrated model of cLBP by combining data from translational research and clinical trials. The success of the BACPAC Research Program requires collaboration and extensive data and resource sharing among its component parts, which demands the development of protocols and standards and commitment to use common data agreed upon by the Consortium.

**Figure 1.**
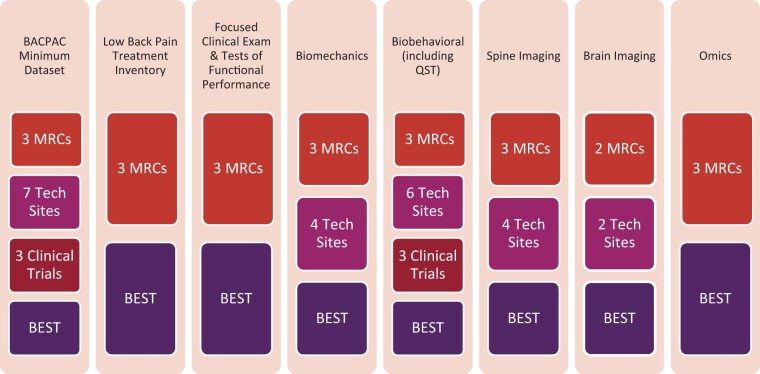
Summary of BACPAC research units contributing to each data domain. MRC= Interdisciplinary Mechanistic Research Center; Tech Sites= Technology Research Sites; BEST= Biomarkers to Evaluate Spine Treatments Trial; QST= quantitative sensory testing.

Content experts representing all components of BACPAC were charged with proposing a case definition for cLBP, developing a BACPAC Minimum Dataset to be obtained for all participants, and identifying outcome measures to be used across all projects. Given the multitude of domains and types of data generated by BACPAC (Figure 1), this effort also included the creation of data standards and the development of guidelines for data governance to facilitate pooled data analyses. BACPAC developed a Data Portal for promoting secure data transfer, storage, and research collaboration across the Consortium.^2^ The aim of the present article is to describe efforts related to data harmonization and accessibility that will facilitate cross-study analyses with HEAL-funded and other pain-related studies after the conclusion of the BACPAC research funding period.

## Methods

### BACPAC data harmonization process

Harmonization efforts across BACPAC were initiated with the NIH’s Common Data Elements (CDE) program.^3^ HEAL defines CDEs as fields describing the data to be collected (eg, identifying specific variables), how to gather the data (eg, participant-reported outcomes), and how the response is represented in a dataset (eg, allowable responses or variable coding).^3^^,^^4^ NIH HEAL Initiative clinical pain research studies are required to collect a core group of CDEs for 9 domains for pain.

The BACPAC Clinical Management Committee (CMC), comprised of clinicians and domain experts, expanded on the HEAL CDEs to include elements specific to cLBP to create the BACPAC Minimum Dataset. The CMC was also charged with harmonizing recommendations for inclusion/exclusion criteria for MRCs.^2^

BACPAC Working Groups (WGs), consisting of researchers throughout the Consortium, were charged with identifying additional domain-specific nonrequired measures and recommendations. Depending on the type of data, harmonization efforts ranged from creating additional CDEs and accompanying data standards to identification of standardized protocols for data collection, file naming, and data submission. Consortium-wide harmonization also included standardized naming of unique subject identifiers, naming and numbering of study visits, and standardization of date variables.

To further advance the NIH’s mission “to facilitate cross-study comparisons and improve the interpretability of findings,”^3^ the DAC was charged with creating a secure central data repository and computing infrastructure for managing data governance and sharing within BACPAC.

### Harmonized BACPAC data

#### BACPAC minimum dataset and nonrequired domains

The Minimum Dataset and Outcome Measures WG was charged with proposing a case definition for cLBP, developing the baseline minimum required CDEs expanding the HEAL requirements, and determining the time periods at which the BACPAC Minimum Dataset should be collected.^2^ The BACPAC Minimum Dataset WG built upon the work of the Research Task Force (RTF) on research standards for cLBP.^5^ The RTF recommended a standard definition for cLBP, as well as minimum demographic and participant-reported descriptive information. The RTF acknowledged that other research groups would expand on this minimum dataset in accordance with their study’s aims, such as for phenotyping purposes. The BACPAC Minimum Dataset includes the RTF recommendations and adds additional questionnaires for broad use across the variety of BACPAC projects, such as phenotyping, behavioral interventions, and imaging studies. The Biobehavioral WG used literature review and discussion-based and consensus decision-making processes to develop a harmonized set of required and nonrequired measures of pain-related and psychosocial/behavioral factors important in chronic pain, with recommendations for specific assessment instruments beyond those included in the BACPAC Minimum Dataset.

#### Low back pain treatments

The CMC was charged with developing data elements to inventory treatments that are commonly prescribed and used for low back pain (LBP), such as surgeries, injections, exercises, spinal adjustments, counseling, and medication. These new data elements were created because the CMC was not able to identify an existing succinct survey for the purpose of finding associations between patient phenotype and responsiveness to LBP treatments that was feasible for observational studies to collect. The content of these data elements was informed by a survey distributed to the leadership of all projects that queried about commonly used and relevant treatments for LBP. The content of the data elements was reviewed and approved during standard CMC meetings. The CMC also recommended time periods to capture this information.

#### Clinical exam, tests of functional performance, and biomechanics data

The Biomechanics and Physical Function WG developed guidelines for harmonizing the clinical exam, tests of functional performance, and biomechanics data, as well as guidelines for submission of nontabular biomechanics data. Objective biomechanical performance measures can provide powerful diagnostic information for musculoskeletal disorders, including cLBP, particularly in the context of simultaneously collected clinical functional outcomes. However, previous cLBP biomechanics studies have used small sample sizes (eg, fewer than 30 subjects per group),^6–9^ which limited the generalizability of the data. Additionally, there have been considerable ambiguity and variability in the goals, designs, and protocols of these cLBP biomechanical studies.^6^^,^^10^ Thus, harmonization of the biomechanics data collected from across the Consortium was deemed to be a key outcome, as was collecting biomechanical, functional, and other outcomes from the same study participants to allow for integration into more comprehensive cLBP phenotypes, with the eventual goal of guiding treatment recommendations.

#### Quantitative sensory testing

The Biobehavioral WG developed guidelines for quantitative sensory testing (QST), which assesses neurophysiological processing of pain. QST can detect alterations in central nervous system processing of sensory information that could be associated with chronic pain.^11^ The QST best practices guidelines were developed by experts from each of the 3 BACPAC MRCs to incorporate tests validated in cLBP to be predictive of outcomes. Efforts were made to balance the feasibility and comprehensiveness of these psychophysical assessments. Decision-making was by consensus among the group of QST experts.

#### Biospecimen collection

In addition to lifestyle and psychosocial contributors, biological biomarkers, such as serum proteins and genetic variants, have been reported to associate with cLBP,^12–17^ which supports the harmonized approach to biospecimen collection and omics analysis described in the Biospecimen Collection and Processing WG paper.^18^ The WG was charged with developing written and video standard operating procedures (SOPs) for collection, storage, processing, and distribution of biospecimens for BACPAC research studies. Included among these are SOPs for biospecimen collection and processing for collection of omics data, including but not limited to genomic data, epigenomic data, transcriptomic data, and proteomic data. To reduce the potential for batch effects and procedural variabilities between collection and processing sites, protocols for biospecimen collection and processing were harmonized as much as possible with those used by the NIH HEAL Initiative and Early Phase Pain Investigation Clinical Network.^19^ Furthermore, the WG was charged with exploring the creation and adoption of a centralized biospecimen processing core and requirements for oversight and quality control.

#### Imaging data

Lumbar magnetic resonance imaging (MRI) is frequently performed in patients with cLBP, but standardization of image acquisition and evaluation across centers is lacking. The Spine Imaging WG was charged with developing consensus-based SOPs for the collection and storage of MRI data and for the reading/grading of images via structured reporting with semiquantitative evaluation and ordinal rating scales. These SOPs aim to facilitate image-based patient phenotyping, to improve understanding of pain mechanisms, and to identify biomarkers in lumbar MRI that inform patient selection for specific treatments.

The Brain Imaging WG was charged with developing functional and structural brain imaging protocols to harmonize data collection across the different sites with brain MRI capabilities. All sites had 3-Tesla scanners and were able to perform acquisition of structural (T1) as well as functional resting state scans. The WG leveraged the extensive experience of harmonizing different scanner brands by using T1 and functional scan acquisition sequences from the Adolescent Brain and Cognitive Development (ABCD) study.^20^ This involved scanning a Biomechanical Information Research Network (fBIRN) phantom and a control traveling human participant at all sites. Harmonization across magnets was made possible by using computer scripts adapted from the ABCD study and applying them to the phantom and human subject data. Standard SOPs and protocols were developed and agreed upon by the Brain Imaging WG. With the harmonization of MRI scanners, the intent was to combine brain T1 and resting state data across sites to improve power and generalizability. This approach is becoming more widely used in large-scale brain MRI studies.

### BACPAC Data Transfer SOP

The Data Sharing, Management, and Standards WG (DSWG) was charged with developing SOPs governing data sharing across BACPAC. The DSWG created the BACPAC Data Transfer SOP to identify harmonized data from each domain, describe the format of harmonized data that should be submitted to the BACPAC Data Portal, outline the transfer schedule, and define how stable data should be identified. The DSWG was charged with developing standards for harmonized tabular data. The Biomechanics and Physical Function WG was charged with developing guidelines for the submission of nontabular biomechanics data. The Systems Biology and Bioinformatics WG was charged with developing guidelines for the harmonization and submission of genomics, transcriptomics, proteomics, metabolomics, and epigenomics data (ie, omics data) from biospecimens. The Brain and Spine Imaging WGs were charged with developing guidelines and standards for the submission of MRI data.

#### Tabular data standards

The DSWG agreed to use a modified version of the Clinical Data Interchange Standards Consortium (CDISC) Study Data Tabulation Model (SDTM),^21^ which is widely used outside of academic settings, to define the standards for the BACPAC Minimum Dataset. The DSWG further identified measures collected across all 3 MRCs in the Consortium to define as broadly collected measures. The DSWG was charged with creating data standards for the broadly collected measures following the same processes as were used for the BACPAC Minimum Dataset. The CDISC SDTM standards are required for submission to the US Food and Drug Administration,^22–25^ and guidelines for their use are freely available and continuously updated to accommodate novel data types. One strong advantage of using the SDTM for tabular data standards is that all of the information in the dataset is contained in the dataset itself and is readable by the user without the need for a code book. For example, data with a coded response (eg, Likert scale) have both the numerical value and its coded character value within the same dataset. Also, these data are self-documenting, meaning that the full question is written out in the dataset, in addition to an (up to) 8-character code for the question. Derived variables are also traceable within the dataset. The DSWG agreed to make small modifications to the CDISC SDTM standards to tailor variables to the data being collected by BACPAC and to reduce the number of datasets that needed to be submitted.

#### Nontabular data standards

The SOP also outlines how data that do not fit in tabular format (eg, omics and imaging) should be harmonized and submitted to the BACPAC Data Portal on the basis of recommendations from the domain experts in the Consortium.

#### Design of the BACPAC Data Portal

The BACPAC Data Portal’s primary purpose is to provide a secure platform for members of the Consortium to conduct collaborative analysis of BACPAC data. The BACPAC Data Portal provides an administrative system for managing the process of collecting data from members, ensuring the integrity of the data, and controlling access to that data for the purposes of analysis and publication. In addition to data management, computing resources are made available to users in the form of virtual machines, which are automatically provisioned with access to the data appropriate to each user. In this way, users can analyze data in a secure environment pre-configured with statistical software. The BACPAC Data Access and Publications Policy outlines the requirements for approval to access the BACPAC Data Portal. For BACPAC affiliates, this includes being listed on the Institutional Review Board Reliance Agreement for the BACPAC Data Portal Protocol and having a Data Use Agreement established between the BACPAC member’s institution and the DAC.

#### Guidelines for metadata and submission to the BACPAC Data Portal

All tabular data submitted to the BACPAC Data Portal are accompanied by a Define-XML metadata file. An advantage of using a modified CDISC SDTM data standard is that Pinnacle 21 provides free software for Define-XML creation,^26^ which uses a standardized specifications file, annotated case report forms, datasets in .csv or .xpt format, and other accompanying documentation and creates an easily navigable hyperlinked file. The metadata are described in greater detail in the Supplementary Material.

## Results

### Harmonized data collection

Each research study collects the BACPAC Minimum Dataset at baseline and at a 3-month visit, where applicable. Additional longitudinal measures include participant-reported outcomes, assessments of functional performance, treatment inventory, and biomechanics data. QST, omics, and imaging data are also collected across a range of studies, including BEST. Several key features of data collection, including the format of date variables, structure of unique subject identifiers, and naming and numbering of visit variables, were harmonized throughout the Consortium. Details are expanded on in the Supplementary Material.

#### BACPAC minimum dataset and nonrequired domains

**Figure 2.**
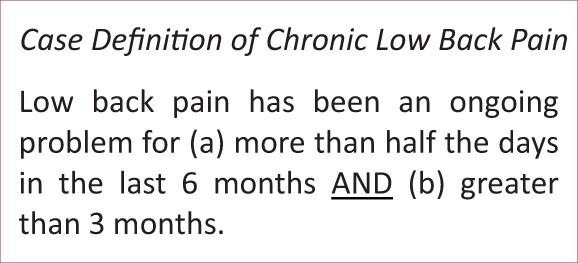
Case definition of cLBP for BACPAC studies.

The BACPAC Minimum Dataset and Outcome Measures WG identified required demographic factors and outcome measures, which are listed in full in a table within another BACPAC Special Issue article by Mauck et al.^2^ BACPAC Minimum Dataset demographic factors to be collected, in addition to the HEAL CDEs, included history of low back surgery, worker’s compensation, legal claims and unemployment due to LBP, whether LBP is more severe than other body pain, and number of persons in the participant’s household.^27^ These back pain–related demographic characteristics and socioeconomic indicators are expected to be important elements in phenotyping and responsiveness to treatments. BACPAC Minimum Dataset outcome measures beyond the HEAL CDEs included LBP-specific pain intensity, duration, and frequency; PROMIS measures of pain interference, depression, fatigue, sleep, and anxiety; and questions about radicular pain, pain somatization, and current opioid use.^27^ Although the HEAL CDEs include 2-item screening instruments for potential depression and anxiety disorders, the PROMIS measures were added because they measure a greater breadth of common depression and anxiety symptoms and are frequently used in the pain literature. Two data elements related to LBP duration and frequency are being collected to characterize the chronicity of LBP^5^ (Figure 2).

All BACPAC projects are required to administer the demographic and outcome measures once. Longitudinal studies are also required to repeat the outcome measures at the 3-month follow-up visits. The rationale for choosing this 3-month follow-up time is 2-fold: First, changes in pain and function with treatment are expected to be evident within 3 months, and second, a 3-month time period is practical for participant recall of treatments and associated outcomes^28^ and is feasible in terms of participant burden and study team resources. In addition to the required 3-month follow-up, longitudinal BACPAC studies complete further follow-ups that are based on their aims.

Required measures for the MRCs include comorbidity assessed by the Charlson Comorbidity Index,^29^ obtained via electronic medical record data and/or self-report, as well as opioid use and dosage in morphine milligram equivalents^30^ obtained via a data collection method at the discretion of project investigators. The MRCs also administer questions about COVID-19 vaccination status, along with symptoms and treatments for participants who have been infected.

Nonrequired measures and outcomes broadly collected by multiple projects include pain characterization with the Michigan Body Map^31^; neuropathic pain assessed by the PainDETECT^32^; the Chronic Pain Acceptance Questionnaire^33^^,^^34^; social role, self-efficacy, and fatigue measured by PROMIS^35–37^; stress measured on the Perceived Stress Scale^38^; fear of movement measured by the Fear Avoidance Beliefs Questionnaire^39^; LBP risk stratification via the STarT Back Screening Tool^40^; and LBP-related disability assessed by legacy questionnaires, such as the Oswestry Disability Index^41^ or the Roland Morris Disability Questionnaire.^42^ The participant-reported outcome recommendations are described in greater detail in the Biobehavioral WG article within the BACPAC Special Issue.^11^

#### LBP treatments

The CMC developed the Treatment Categories Questionnaire to inventory common LBP treatments.^43^ Administration of this questionnaire is required for the MRCs once per month during the first 6 months for each study participant. Monthly administration helps participants recall their LBP treatments. This information will be used for phenotyping based on treatment response. Treatment elements include surgeries, injections, and medication for LBP. Medication data are collected, specifically with regard to the start of or change in use of opioids, selective serotonin reuptake inhibitors and serotonin and norepinephrine reuptake inhibitors, gabapentin or pregabalin, tricyclic antidepressants, and nonsteroidal anti-inflammatory drugs. Data are also collected for treatments provided by physical/occupational therapists and chiropractors, including adjustment/manipulation, supervised active exercises, and passive modalities such as ultrasound. Additional questionnaire items ask about unsupervised exercise, acupuncture, mental health therapy or counseling, mindfulness and other relaxation approaches, and diet or weight loss programs. To obtain broad information on commonly used LBP treatments, treatments prescribed by a provider and initiated by the study participants are both collected. Telehealth information is obtained to capture changes in treatment delivered during the COVID-19 pandemic. The reference period for the questions is “within the last month” to align with the frequency of administration.

#### Clinical exam, functional performance, and biomechanics

The Biomechanics and Physical Function WG harmonized the clinical examination for the neurological screening tests of sensation, deep tendon reflexes, myotome function, and seated slump test. The following were harmonized across all MRCs: measures of range of motion, including spine flexion and extension and hip internal rotation; administration of the prone instability test; repeated movements of the lumbar spine to observe changes in signs and symptoms; functional performance tests of gait speed and endurance; and the 5-times sit-to-stand tests. The following were harmonized in 2 out of 3 MRCs: the plantar reflex (Babinski) test, the Beighton Score for generalized joint hypermobility, the lumbar segmental mobility test, active and passive straight leg raise tests, the pain provocation tests for the sacroiliac joint, tests for hip extension and abduction strength with the use of a dynamometer, the active sit-up endurance test, and the single leg-stance time. The MRCs shared SOPs and scoring rubrics for all harmonized tests. Harmonization of the tests and standardization of the procedures within each test facilitate comparisons of the different MRC samples and pooling of data for more powerful analyses with regard to the phenotyping goals of BACPAC.

In addition to the clinical examination, the Biomechanics and Physical Function WG harmonized a suggested series of static, dynamic, and functional kinematics measures collected during the accomplishment of subject movements. Static measures include anthropometry metrics (height, weight) and demographic metrics (sex, age). Dynamic measures include position, velocity, and acceleration over time while performing the designated subject movements. The subject movements were not completely harmonized because of a lack of consensus in the literature on which movements are most likely to have diagnostic capabilities. Thus, the Consortium settled on a requirement that each site collect movement activities that demonstrated functional metrics, including strength, endurance, power, symmetry, coupling, and balance. Activities common to most sites include measuring kinematics during single-axis movements (flexion-extension, axial rotation, lateral bending), multi-axis movements (flexion-rotation, flexion-lateral bending), and functional movements (“up and go,” “box lift”). Each of these common activities is routinely performed as part of both diagnostics and treatment in clinical practice. These measures are also common to research studies because they probe the functional metrics described previously (strength, endurance, power, symmetry, coupling, and balance). Deficiencies in these metrics might define phenotypes in individuals with cLBP that could be responsive to particular therapeutic treatments. The technologies used to collect this information are described in greater detail in the Biomechanics and Physical Function WG article within the BACPAC Special Issue.^44^

#### Quantitative sensory testing

The Biobehavioral WG best practices guidelines recommend pressure pain threshold and temporal summation tests, with the option of conditioned pain modulation, and provide protocols for these tests that were distributed throughout the Consortium.^45^ The 2 recommended tests, pressure pain threshold and temporal summation, are harmonized in BACPAC to support comparison with other studies that use these existing standard protocols. For projects that include conditioned pain modulation, BACPAC will address an existing gap in the literature with regard to optimal procedures and techniques for conditioned pain modulation by providing data on large numbers of patients collected with slight variations in conditioned pain modulation methods. The QST methods and rationale are described in greater detail in the Biobehavioral WG article within the BACPAC Special Issue.^11^

#### Biospecimen collection

The Biospecimen Collection and Processing WG defined and established the process for collection and analysis of key biospecimens for which a relationship with cLBP has been identified. The omics covered will include genomics (DNA), epigenetics (DNA), proteomics (protein), transcriptomics (RNA), and microbiomics (16 s rRNA). Whole blood and saliva will be collected at all MRCs. The University of Pittsburgh will additionally collect urine, stool, and spine tissue samples. The University of California San Francisco will also collect stool. The University of California San Francisco will be the only site that uses whole-blood samples for DNA (PAXGene analysis). Harmonization by the WG and the rationale for collection of these biospecimens are described in full in another article in the BACPAC Special Issue.^18^

#### Imaging data

The Spine Imaging WG recommended a noncontrast MRI exam with standard clinical pulse sequences on 3-Tesla or 1.5-Tesla MRI scanners.^46^ Specifically, the exam includes imaging with T1- and T2-weighted sequences covering the lumbar spine and sacrum with the use of a minimal pulse sequence protocol with the following sequences: sagittal T2-weighted fast spin-echo (FSE) sequence with fat saturation, sagittal T1-weighted FSE sequence without fat saturation, and axial T2-weighted FSE sequence without fat saturation. If feasible, the minimal pulse sequence protocol may be supplemented with the following additional recommended sequences: sagittal T2-weighted FSE sequence without fat saturation, sagittal T1-weighted FSE sequence without fat saturation, coronal T1-weighted FSE sequence without fat saturation, axial T1-weighted FSE sequence without fat saturation, and 3-dimensional (3D) T2-weighted FSE sequence with fat saturation for neurography. The Spine Imaging WG also proposed recommended ranges for the pulse sequence parameters (eg, echo time, relaxation time) that comply with the MRI infrastructure at the various BACPAC imaging sites.

In addition to recommending a standardized MRI exam, the Spine Imaging WG also developed a qualitative/semiquantitative scheme for evaluating the images that is based on several established ordinal rating scales for structured reporting of lumbar spine pathologies. This scheme incorporates the different spinal structures and related grading of the following pathologies: Modic-type endplate changes, endplate defects, intervertebral disc changes, facet joint and sacroiliac joint changes, and stenosis. The MRI acquisition protocol, pulse sequence parameters, and image evaluation recommendations are published in a separate article within the BACPAC Special Issue.^46^

Brain imaging was calibrated across 6 different sites: University of Michigan, Massachusetts General Hospital, University of California San Francisco, University of California Davis, University of California Irvine, and University of California San Diego. All sites used either GE or Siemens 3-Tesla scanners and collected T1 and resting-state data with MR pulse sequences adapted from the ABCD study. Once the fBIRN phantom had been scanned at each site, data were uploaded to the University of Michigan for quality control. These metrics included signal-to-noise ratio, root mean square, drift, full width half maximum, mean ghosting, and others. All magnets displayed parameters within the normal range reported by the ABCD collaborative. After scanning of the fBIRN phantom, a single healthy human participant also underwent the same T1 and resting-state scans. These data are currently being analyzed. As all sites had successful fBIRN phantom data, study participants were allowed to be imaged, with sites performing quality control images of their fBIRN phantom at regular biweekly intervals to test for scanner drift or other aberrations that might impact brain imaging data.

### Modified SDTM CDISC standards

The CDISC SDTM provides a standard for organizing and formatting data to streamline the processes of data collection, management, analysis, and reporting.^21^ BACPAC members agreed on a version of these standards that was modified to meet the specific needs of back pain research. Six common domains were used to define standards for demographic, subject characteristic, participant-reported outcome, LBP treatment, functional test, and biomechanics-derived data. Each domain (ie, dataset) within the BACPAC data standards was assigned a 2-character abbreviation that is incorporated into the dataset and variable naming conventions per the CDISC SDTM standard, as shown in Table 1.

**Table 1.** The set of domains (ie, datasets) with BACPAC-developed tabular data standards^47^

CDISC domain(s)	Data description
DM—Demographics / SC—Subject Characteristics	Baseline demographics and participant characteristics
QS—Questionnaires	Participant-reported outcome (questionnaire) data
EX—Exposures	Longitudinal treatment information
FT—Functional Tests	Physical function and QST
BM—Biomechanics	Biomechanics tabular data

CDISC = Clinical Data Interchange Standards Consortium; QST= quantitative sensory testing.

Table 2 shows variable names common to multiple datasets. Aside from small modifications discussed in this section, the demographics dataset follows established CDISC standards and code lists. BACPAC uses the Subject Characteristics (SC) domain for baseline characteristics data from the BACPAC Minimum Dataset that do not fit into established standards in the Demographics (DM) domain. The structure of the data is such that the DM dataset has 1 row per participant, whereas all other datasets are in long format in which each participant has 1 row per unique test (ie, measure) per time point. For example, in Questionnaires (QS) datasets from longitudinal research studies within BACPAC, each participant will have 1 row per measure from the BACPAC Minimum Dataset for the baseline visit (Week 0) and 1 row for the 3-month visit (Week 12). Functional Tests (FT) datasets might have additional rows per measure if the test was performed for multiple repetitions or with multiple body parts.

**Table 2.** Study Data Tabulation Model (SDTM) variable names and descriptions

Type of variable and variable name	Description
Identifier variables	
STUDYID	Study ID
USUBJID	Unique subject ID
--SEQ	Variable to identify unique observations in a dataset
Timing variables	
VISIT	Visit name
VISITNUM	Visit number
--DY	Study day of finding
--DTC	Date/time of finding
--EVLINT	Evaluation interval
Grouping and synonym qualifiers	
--CAT	Category (grouping qualifier)
--SCAT	Subcategory (grouping qualifier)
--TEST	Name of measurement, test, or examination (synonym qualifier)
--TESTCD	Abbreviated test code (grouping qualifier)
Result qualifiers	
--STRESC	Character result or finding in standard format
--STRESN	Numeric result or finding in standard format
--ORRES	Result or finding in original units
Record qualifiers	
--DRVFL	Derived flag
Variable qualifiers	
--STRESU	Standard units of result or finding
--ORRESU	Units of original result or finding

Tabular data associated with the QS, FT, and Exposure (EX) domains may have category (--CAT) and subcategory (--SCAT) values assigned to a given measure or test. Categories for participant-reported outcome measures were harmonized across the BACPAC Minimum Dataset and broadly collected measures. Subcategories (QSSCAT values) were defined as the case report form name (eg, QSSCAT = PROMIS Emotional Distress—Anxiety), as shown in Table S2. For the FT domain, the FTCAT values are the full names of the case report forms that describe the physical function tests.

Values of variables ending in --TEST are the full name of the question or measure from the case report form, up to 100 characters. Values of variables ending in --TESTCD are the corresponding unique (up to) 8-character code for the measure. Where possible, existing --TESTCD values were used. For example, many PROMIS measures have existing --TESTCD values on case report forms freely available via online sources.^48^ In this way, the data are self-documenting, and a code list is not needed to identify the measure being evaluated.

Another example of how the datasets are self-documenting is the use of both original result values (eg, --ORRES = **or**iginal **res**ult) with original units (eg, --ORRESU = **or**iginal **res**ult **u**nits) and standardized result values (eg, --STRESC = **st**andardized **res**ult **c**haracter and --STRESN = **st**andardized **res**ult **n**umeric) and standardized units (eg, --STRESU = **st**andardized **res**ult **u**nits) in the same dataset. In the clinic, height and weight might have been reported in inches and pounds, which is retained in the dataset (SCORRES, SCORRESU), along with the converted measurements in metric units (SCSTRESC, SCSTRESN, SCSTRESU), as shown in Table S3. As a rule, numeric result values (--STRESN) are also recorded as the character result values (--STRESC) if no corresponding character value exists.

Modifications were made to the CDISC SDTM data standards to minimize the need for supplemental datasets and better meet the specific needs of BACPAC. For example, the RACEMULT variable was added to the DM dataset to list participants’ multiple self-identified races. Although the variable corresponding to gender identity was standardized for all HEAL-funded studies, BACPAC investigators requested the ability to use more inclusive gender identity response options. This resulted in a recommended set of expanded response options to use during data collection and instructions on how to map those responses to the HEAL-required responses (Table S4). Additional details about the BACPAC-modified CDISC SDTM standards are located in the Supplementary Material.

### BACPAC Data Portal

The BACPAC Data Portal was developed via a collaboration with Microsoft and the BACPAC DAC team at the University of North Carolina Chapel Hill’s Collaborative Studies Coordinating Center. It is a secure cloud-based central data repository and computing infrastructure hosted in Microsoft Azure for researchers to access and conduct analyses on various types of data collected by or acquired for the BACPAC Research Program. Authenticated users access the BACPAC Data Portal through a Web browser, where they can submit their study's data, browse data hosted on the BACPAC Data Portal, upload or download code and output to their personal and project workspaces, and access Linux and Windows virtual machines with pre-installed analysis tools, such as Python, R, SAS, STATA, and MATLAB, and high-performance computing capabilities. Users can install libraries from CRAN and PIP and can import Docker containers to support unanticipated software requirements.

Beyond the security and software requirements, a few critical features include streamlined user management functionality, data organization and versioning, and the ability to transfer large files. The ability to upload large files (tens of gigabytes in size) is usually a challenge for many online/cloud systems, as data are often too large to transmit over standard network connections. Many projects resort to sending the physical media to storage centers. The BACPAC Data Portal has a fairly sophisticated and very secure mechanism (SAS keys with Azure Storage Explorer) for researchers to upload large files (up to 100 gigabytes per file) and folders that contain 1 or more files. In addition, data versioning and user management are often time consuming and unwieldy. The BACPAC Data Portal team developed a solution that allowed DAC administrators to invite users and update data access from within the BACPAC Data Portal interface by using research site– and project-based access controls. Similarly, the management of data versioning was automated through the BACPAC Data Portal with a mechanism that also provides researchers with appropriate access to data that are mounted within their virtual machines in standard folder structures.

Data files and related documentation submitted to the BACPAC Data Portal must adhere to the formats and requirements described in the BACPAC Data Transfer SOP. Once data have been submitted to the BACPAC Data Portal, the DAC verifies that all received data and documentation conform to the BACPAC data standards and approves the data for use on the BACPAC Data Portal. At that point, authorized users gain read-only access to the new data (or a new version of existing data) to use for their approved research activities. The Supplementary Material provides a more detailed description of the BACPAC Data Portal.

### Accompanying documentation

Annotated case report forms (Figure S3), metadata specification files (Figure S4), and simulated datasets exemplifying the data standards (Table S3) were made available for all research groups to use and modify to serve the needs of their studies. Annotated case report forms outline which variables and which variable values correspond to questions on the form to facilitate adoption of the data standards. The DAC also provided guides for data standards and Define-XML creation. Free Pinnacle 21 software was used to generate Define-XML files (Figures S5 and S6). These files provide hyperlinks to annotated case report forms, datasets, code lists, definitions of algorithms, and all other accompanying documentation.

## Discussion

Harmonization efforts within the BACPAC Research Program resulted in the development of the BACPAC Minimum Dataset, recommendations for domain-specific data collection, SOPs for data collection and reporting, data standards for harmonized data elements, and the BACPAC Data Portal for collaborative research. Successful implementation across 14 different research units required extensive input from and coordination, collaboration, and cooperation among BACPAC members. These recommendations complement the CDEs required of all HEAL studies and will facilitate integration of demographic and pain-related data from 5000 observational study participants at MRCs and hundreds of additional participants from BACPAC Clinical Trial Centers and Tech Sites. Integrating data from harmonized measures improves the efficiency of conducting analyses because of increased sample size, and pooling participant data from sites nationwide improves generalizability. Research proposals for integrated analyses of BACPAC data are currently being drafted, and the unique structure of the Consortium studies and their data motivate and facilitate the development of statistical and analytical methods in the fields of precision medicine, causal inference, machine learning, translational science, and bioinformatics.

Harmonization of measures across the various data domains serves to address multiple aims of BACPAC. Pooled analysis of demographic and descriptive measures from the BACPAC Minimum Dataset allows for characterization of cLBP patient phenotypes and identification of subgroups for further analysis. Data from additional domain recommendations, as well as deep phenotyping of 1800 of the observational study participants, allow for analysis and investigation of additional psychosocial, genetic/biological, imaging, and biomechanical biomarkers that characterize the disease course in cLBP and might be predictive of treatment response. Harmonized treatment information and outcome measures from observational studies and clinical trials inform the precision medicine aim of evaluating the right treatment for the right patient at the right time. Integrated analysis of BACPAC data will facilitate translation to clinical practice more easily than would examining data from individual studies.

The data harmonization of BACPAC reflects a concerted effort to maximize the integrability of data collected in its diverse research program and to minimize the often-substantial time and learning curve for downstream consumers of its data. However, the initial work of putting the data into SDTM standards was not without significant effort by research unit programmers who work with unique and customized data collection systems and databases. For example, several universities used a customized electronic data capture system, whereas others used REDCap. The format of the raw data available to programmers at each site could require extensive recoding of variables and creation of new variables. The DM domain, which is the only dataset in wide format, was noted as being the easiest to convert to the required format, but other domains, which are in long format, require complex dataset restructuring.

Programmers and analysts noted that the most helpful resources were the annotated case report forms and the simulated datasets, which allowed mapping of the annotations on their site-specific case report forms to the annotations for the required format. Sites could then check that their output resembled the structure and formatting of the simulated datasets. In the future, other research programs using the BACPAC WG recommendations for data collection can use the annotated case report forms developed by BACPAC for ease of data conversion or can elect to program initial data capture by using the unique test codes developed by BACPAC. Future work for the Consortium includes publishing more detailed guides for use of the data standards and a GitHub repository for SAS and R programs that are used to clean and check datasets to ensure they are in the required format.

Ultimately, these efforts will contribute to the ease and accessibility of future analyses of these invaluable datasets, as well as analysis and integration of the data with future studies after the initial BACPAC studies are completed. NIH HEAL Initiative grant requirements dictate that all studies be FAIR (findable, accessible, interoperable, and reusable). These requirements were adopted in response to the urgency of the nationwide opioid epidemic and the need for immediate use of HEAL data for clinical translation. The data-sharing requirements were unique to HEAL at the time of grant funding but are becoming the standard for all NIH-funded studies.^49^ The development of Consortium-wide data standards accelerates compliance with HEAL data-sharing requirements. BACPAC data harmonization efforts and data standards and the BACPAC Data Portal serve as an innovative model for data integration that could be used as a framework for other consortia with multiple decentralized research programs.

## Supplementary Material

pnad008_Supplementary_Data
