# Supplementary material for: The Back Pain Consortium (BACPAC) Research Program Data Harmonization: Rationale for Data Elements and Standards
Source: Pain Med. 2023 Jan 31;24(Suppl 1):S95–S104. doi: 10.1093/pm/pnad008 (PMC11288398; doi:10.1093/pm/pnad008)
Supplement: pnad008_Supplementary_Data [file pnad008_supplementary_data.docx]

**Supplemental Material**

The BACPAC Research Program Data Harmonization: Rationale for Data Elements and Standards

# Key Features of Consortium-Wide Data Harmonization

Several key features of the data were harmonized across the Consortium. For example, the format of BACPAC unique subject IDs follows [Study ID] – [Site ID] – [Participant ID] where each study has a unique character name, the site IDs are systematically assigned, and the participant ID is a unique five-digit number with leading zeros.

Instead of each study having their own numbering scheme for visit number, the character variable VISIT and numeric variable VISITNUM were harmonized across the Consortium as shown in Table S1. Data standards describe the naming and numbering convention for unplanned visits and visits more frequent than a weekly interval.

Table S1. Description of harmonized visit variables^1^.

| Nominal Timing of Collection | Visit Description  (VISIT) | Visit Number (VISITNUM) |
| --- | --- | --- |
| X weeks prior to protocol-specific reference visit | Week -X | -X |
| Protocol-specific reference visit (e.g., randomization visit) | Week 0 | 0 |
| 7 weeks post baseline | Week 7 | 7 |
| 3 months post baseline | Week 12 | 12 |
| 6 months post baseline | Week 26 | 26 |
| 30 weeks post baseline | Week 30 | 30 |
| 1 year post baseline | Week 52 | 52 |
| 2 years post baseline | Week 104 | 104 |

BACPAC data standards follow ISO8601 formatting for date and duration variables (e.g., birth date, date of findings, duration of sleep, etc…), which is an internationally recognized standard.

# Tabular Data Standards Harmonization

Each domain (i.e., dataset) within the BACPAC data standards is assigned a two-character abbreviation that is incorporated into the dataset and variable naming conventions per the CDISC SDTM standard (e.g., DM=Demographics, QS=Questionnaire). Refer to Table 1 in the main paper for the full listing.

Tabular data associated with the Questionnaires (QS), Functional Tests (FT), and Exposure (EX) domains may have category (--CAT) and subcategory (--SCAT) values assigned to a given measure or test. Categories for participant-reported outcome (PRO) measures were harmonized across the BACPAC Minimum Dataset and broadly collected measures. Subcategories (QSSCAT values) were defined as the form name (e.g., QSSCAT=PROMIS Emotional Distress – Anxiety) as shown in Table S2.

These data standards eliminate the need for external code books because datasets contain both character and corresponding numeric variables for all measures.

Table S2. Categories and subcategories for BACPAC Minimum Dataset items.

| Category (QSCAT) | Subcategory (QSSCAT) |
| --- | --- |
| Pain Duration and Frequency | Low Back Pain Duration and Frequency |
| Pain Location | Radicular Low Back Pain |
| Widespread Pain | Widespread Pain Inventory |
| Pain Somatization | Abbreviated Pain Somatization |
| Pain Catastrophizing | Pain Catastrophizing Scale |
| Pain Intensity | Low Back Pain Intensity |
| Pain Intensity | Relative Severity of Low Back Pain |
| Pain Intensity and Interference | PEG |
| Pain Interference | PROMIS Pain Interference |
| Physical Function | PROMIS Physical Function |
| Sleep | PROMIS Sleep Disturbance |
| Sleep | Sleep Duration |
| Depression | PHQ |
| Depression | PROMIS Emotional Distress - Depression |
| Anxiety | GAD |
| Anxiety | PROMIS Emotional Distress - Anxiety |
| Patient Satisfaction | Patient Global Impression of Change |
| Substance Use | TAPS |
| Opioid Use | Current Opioid Use |

Table S3. Example of original result (SCORRES) and units (SCORRESU) variables along with standardized character (SCSTRESC) and numeric (SCSTRESN) variables with standardized units (SCSTRESU), for height and weight measurements in subject characteristics (SC) domain^1^.

# Expanded Options for Gender Identity

The BACPAC Data Sharing, Management and Standards Working Group (DSWG) recommended the use of expanded gender identity options during data collection. While the BACPAC Minimum Dataset itself remained as was approved by the BACPAC Steering Committee, research units were able to use an expanded list of gender identity options as recommended by the DSWG. The requirement was set that all gender identity data submitted to the BACPAC Data Portal and HEAL repository would be mapped to the original BACPAC Minimum Dataset / HEAL categories to ensure the HEAL common data element definitions were maintained (see Table S4 for the recommended options and mapping).

Table S4. Expanded options for Gender Identity as part of the BACPAC Minimum Dataset.

| **Original BACPAC Minimum**  **Dataset (HEAL) Options** | **DSWG** **Recommended Expanded Options and Mapping to**  **Original BACPAC Minimum Dataset (HEAL) Categories** | | |
| --- | --- | --- | --- |
| Gender Identity Coded Value:  1. Male  2. Female  3. Unknown  4. Other, specify: __________ | Gender Identity Coded Value:  1. Male  2. Female  3. Transgender man  4. Transgender woman  5. Genderqueer or gender nonconforming  6. Unknown  7. Other, specify: __________ | =>  =>  =>  =>  =>  =>  => | HEAL Coded Value  Male  Female  Male  Female  Other  Unknown  Other |
|  |  |  |  |

# BACPAC Data Portal

The BACPAC Data Portal is a web-based platform which manages the entire lifecycle of BACPAC data from submission of study data to analysis on a secure computing platform.

Key Features:

- An open-source web-based application developed in collaboration with Microsoft to provide researchers with an easy-to-use interface for accessing data and compute resources hosted by Microsoft Azure.
- Provides a "one stop" collaborative hub with computing resources, including high performance computing, personal and project workspaces for collaboration and analyses, and secure storage of data hosted on the BACPAC Data Portal.
- Supports the secure transfer of large files (up to 100 GBs per file) directly from a researcher's local computer.
- Manages data versioning through its customized data transfer process and provides researchers with standard directory structures within their virtual machines (VMs) to easily identify and access current or archived versions as is needed for their analyses.
- Output such as derived data files, results, and program logs can be written to the user's own workspace or a shared project workspace from within the VMs and approved output files can be downloaded from the web browser interface.

Submitted data files and related documentation must adhere to the formats and requirements described in the BACPAC Data Transfer SOP. Once submitted, the BACPAC Data Portal team verifies all received data and documentation conform to the BACPAC data standards and approves for use on the BACPAC Data Portal. At that point authorized users gain read-only access to the new data or a new version of data to utilize for their approved research activities. All data access is approved for each user according to the BACPAC Data Access and Publications policy.


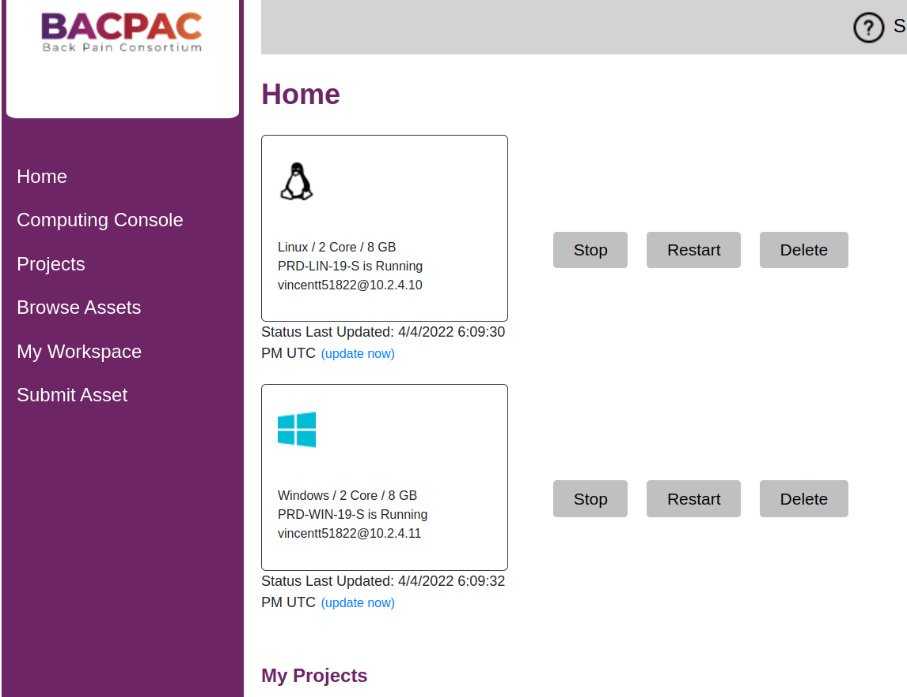


Figure S1. The BACPAC Data Portal home page, where users can view their computing resources and project membership. The menu to the left allows users to explore BACPAC data, manage their personal or project workspaces, and interact with their virtual machines^1^.


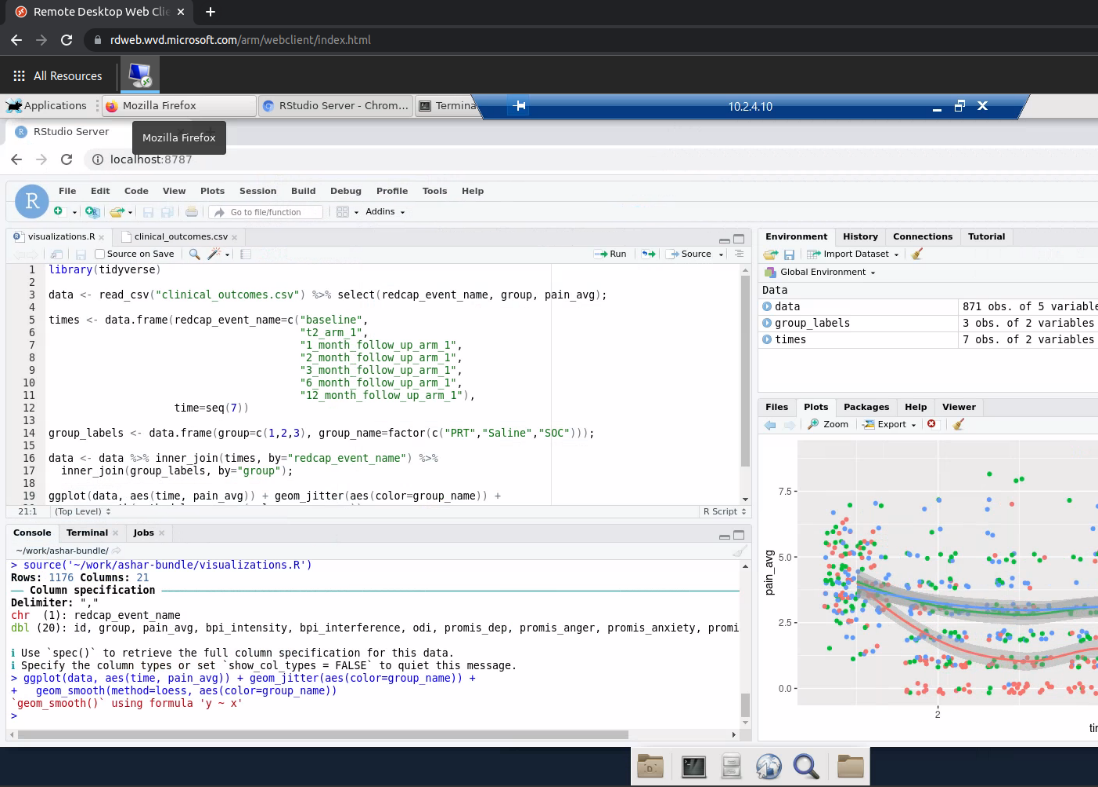


Figure S2. BACPAC VMs are pre-configured with common statistical software and configured for authorized users to gain read-only access to data they have been approved to utilize for their approved research activities. While VMs are locked out of most of the internet for security and data integrity reasons, users may upload Docker containers to support arbitrary development environments^1^.

# Data Documentation and Submission

Figure S3 shows a section of the case report form for the BACPAC Minimum Dataset. Case report forms were annotated for all required measures and some non-required measures to facilitate conversion into the BACPAC data standards. Annotated case report forms (aCRFs) show the data domain (e.g., QS=Questionnaires in Figure S3), category, subcategory and evaluation interval if applicable, identify the test code for each measure, and display the standardized numeric and character result values.


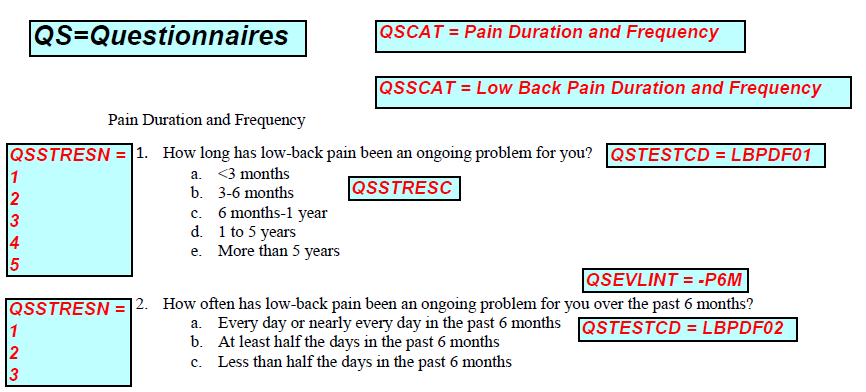


Figure S3. Annotated case report form example.

All tabular data are required to be submitted with a specifications file in a standardized format. The specifications file is an Excel workbook that organizes study information (Study), dataset metadata (Datasets), variable descriptions (Variables), code lists (Codelists), and supplemental documentation (Documents) into different worksheets. The WhereClauses and ValueLevel worksheets map code lists to the questions and variables to which they correspond. Members of the DSWG developed example specifications files for each data domain that research sites can customize as appropriate.


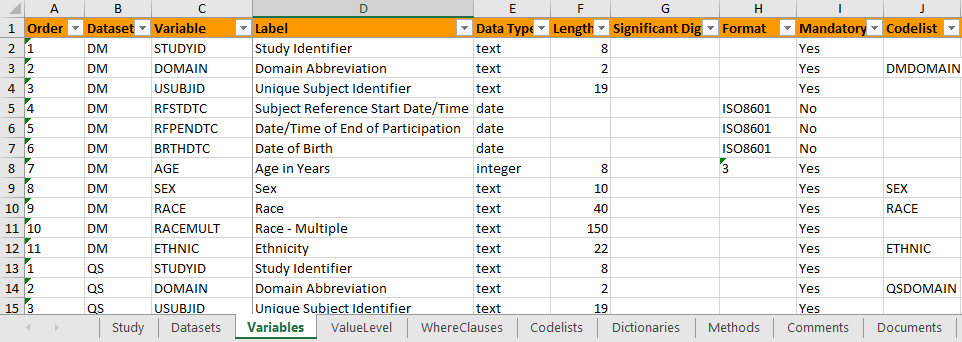


Figure S4. Variables worksheet of standardized specifications file describes variables for each dataset^1^.

Free Pinnacle 21 software uses the standardized specifications file and allows fast and easy creation of Define-XML files, which provide hyperlinks to the metadata for all submitted datasets. As shown in Figure S5, the sidebar on the left contains hyperlinks to annotated case report forms, descriptions of variables in each dataset, and code lists. Tabular datasets may be submitted as SAS transport (.xpt) files or comma separated values (.csv) files. The ability to work with and submit datasets without the need for costly proprietary software is a desirable feature of BACPAC data standards, necessitated by the diversity of data management and analysis methods of research sites and downstream consumers of BACPAC data.


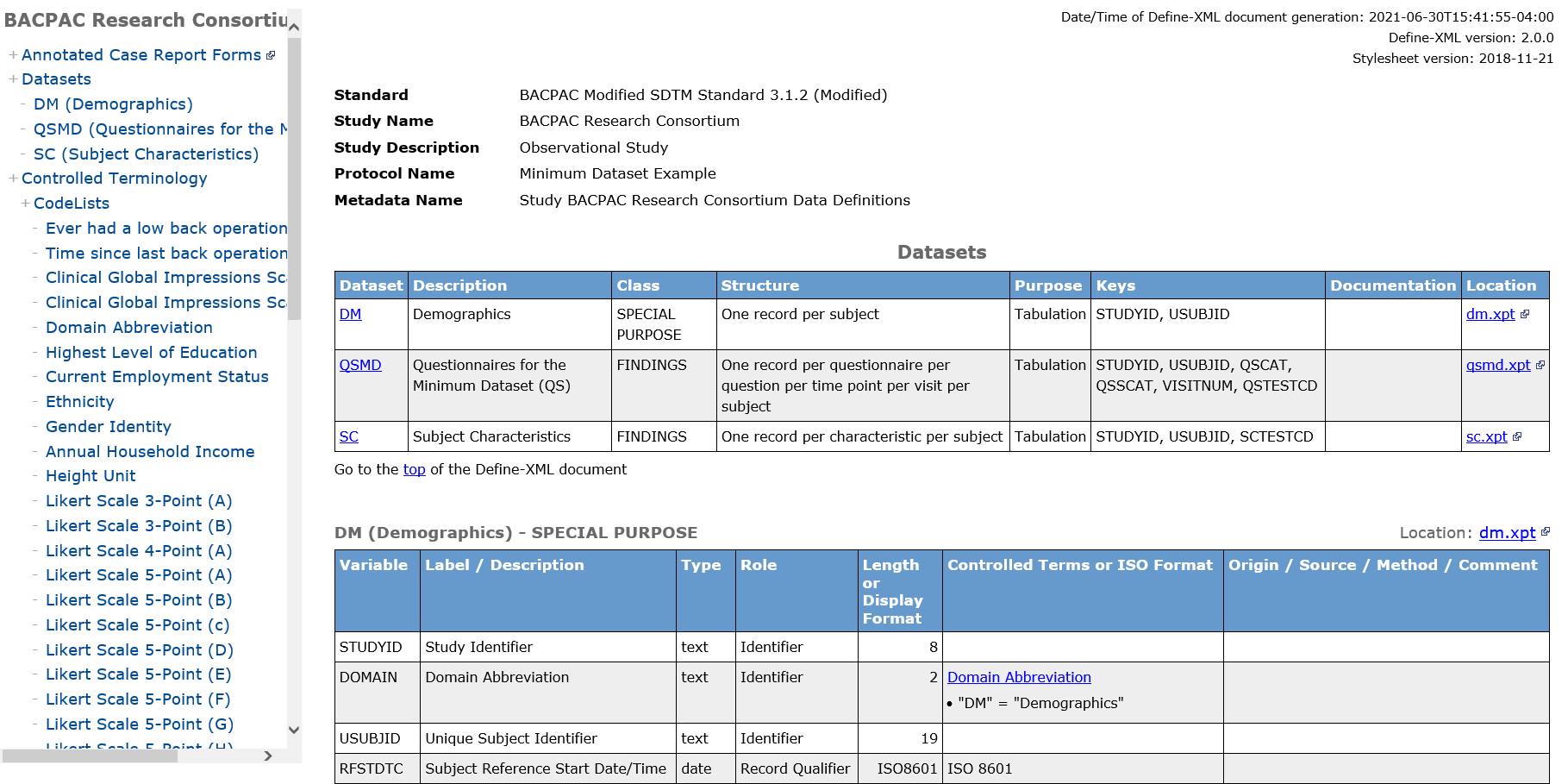


Figure S5. Top of Define-XML file for BACPAC Minimum Dataset example^1^.


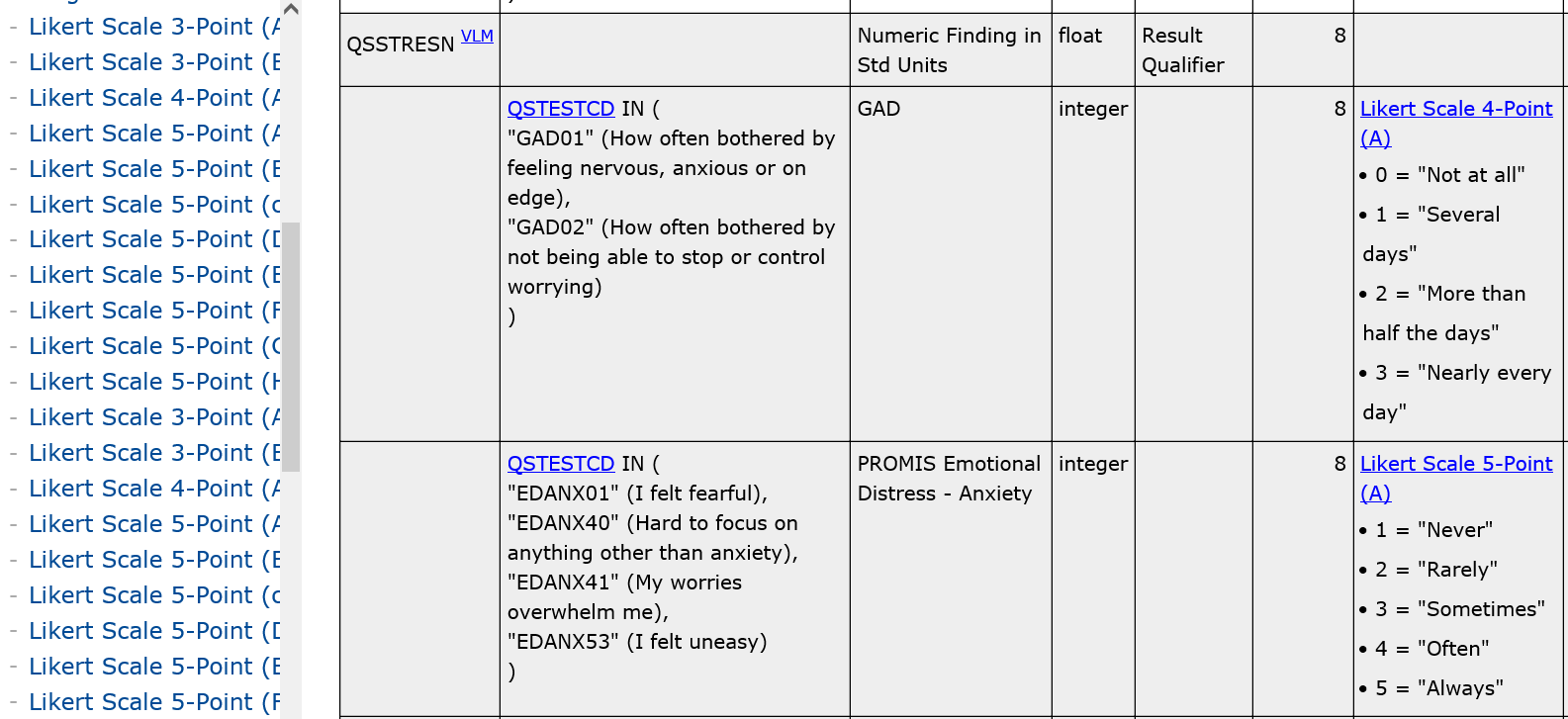


Figure S6. Metadata for each domain contains hyperlinks to code lists and shows how each question maps to each code list for character and numeric values of the code list^1^.

Availability of simulated example datasets for each data domain and guides for data standard usage and Define-XML creation facilitates adoption of standards for harmonized and non-harmonized data.

**References**

1. Batorsky A, Toups V, McCumber M, Patterson CG, Psioda MA. BACPAC Data Portal and Harmonized Data Standards Facilitate Data Integration Across Multiple Back Pain Studies. Presented at: Third Annual NIH HEAL Initiative Investigator Meeting; April 2022; Virtual.
